# A non-cell autonomous mouse model of CNS haemangioblastoma mediated by mutant KRAS

**DOI:** 10.1038/srep44899

**Published:** 2017-03-21

**Authors:** Leyuan Bao, Osama Al-Assar, Lesley F. Drynan, Mark J. Arends, Pam Tyers, Roger A. Barker, Terence H. Rabbitts

**Affiliations:** 1 Weatherall Institute of Molecular Medicine, MRC Molecular Haematology Unit, University of Oxford, Oxford, OX3 9DS, UK; 2 MRC Laboratory of Molecular Biology, Francis Crick Ave, Cambridge CB2 OQH, UK; 3 Centre for Comparative Pathology & Division of Pathology, University of Edinburgh, Institute of Genetics & Molecular Medicine, Crewe Road, Edinburgh, EH4 2XR, UK; 4 John van Geest Centre for Brain Repair, Forvie Site, Robinson Way, Cambridge CB2 0PY, UK

## Abstract

Haemangioblastoma is a rare malignancy of the CNS where vascular proliferation causes lesions due to endothelial propagation. We found that conditionally expressing mutant *Kras*, using *Rag1-Cre,* gave rise to CNS haemangioblastoma in the cortex and cerebellum in mice that present with highly vascular tumours with stromal cells similar to human haemangioblastomas. The aberrant haemangioblastoma endothelial cells do not express mutant *Kras* but rather the mutant oncogene is expressed in CNS interstitial cells, including neuronal cells and progeny. This demonstrates a non-cell autonomous origin of this disease that is unexpectedly induced via *Rag1-Cre* expression in CNS interstitial cells. This is the first time that mutant RAS has been shown to stimulate non-cell autonomous proliferation in malignancy and suggests that mutant RAS can control endothelial cell proliferation in neo-vascularisation when expressed in certain cells.

Mouse models of human cancer allow for a better understanding of the cell of origin, the disease process and for therapeutic testing. Many different brain tumours are found in humans of which about 1% are haemangioblastomas that are slow growing and are non-invasive caused by vascular endothelium expansion within brain tissue^1^. They can be found in the brain stem, cerebellum (the most common form) and in the cerebrum and associated with von Hippel Lindau syndrome^2^ where it presents in a more malignant form.

There are currently no mouse models of haemangioblastoma in the CNS. We discovered a mouse model of haemangioblastoma in which endothelial cell proliferation occurs to cause deleterious lesions in the brain when we implemented activation of a conditional form of floxed mutant Kirsten *Ras (KRas*) with glycine 12 mutation to valine (KRasG12V)^3^ using the VDJ recombinase activating gene 1 (*Rag1*) to express Cre recombinase^4^. This was unexpected since the conditional *KRas* allele was activated by *Rag1-Cre* expressed in the brain and because the endothelial cells involved do not themselves express the mutant allele of *KRas,* signifying a non-cell autonomous proliferation causing this disease.

## Materials and Methods

### Mouse strains and haemangioblastoma pathology

Floxed *KRasG12V* mice^3^ were crossed with *Rag1-Cre* knock in mice^4^ to generate mice carrying both alleles. When abnormal phenotypes developed, mice were sacrificed by terminal anaesthesia and perfusion fixation. Whole brains were dissected carefully and fixed in 4% paraformaldehyde for 24 hours and equilibrated in 20% sucrose in PBS-0.1%azide at 4 ^o^C overnight. 20 uM sections were prepared on a freezing microtome and stained either with cresyl violet or haematoxylin and Immunohistochemistry carried out with anti-CD31 at 1/20 dilution of rabbit anti-CD31 (Abcam-ab28364) with citric acid antigen retrieval method. All experiments with mice were conducted under a Home Office Personal License and with agreement from the University of Oxford Animal Welfare and Ethical Review Committee.

### Reporter gene expression analysis

*Rag1-Cre* mice were bred with *ROSA-tdTomato* reporter mice^5^ and 30 day old heterozygous pups were perfusion fixed with 50 mL 10% buffered formalin after clearing the blood with 50 mL of ice cold PBS supplemented with 1 mM EDTA and the brain removed. Post- dissected whole brains were fixed in ice cold 10% buffered formalin for one hour and submerged in 25% sucrose/PBS overnight. Brains were mounted in tissue freezing medium (Fisher Scientific) and snap frozen on dry ice assisted by freezing aerosol (VWR). Sections were subsequently cut using a Leica cryostat system at 10 μm thickness and allowed to adhere to Superfrost Plus slides (Fisher Scientific) at 37 °C for one hour. Sections were stored for short term at 4 °C before further processing. For microscopy, sections were first mounted in ProLong Gold containing DAPI (Life Technologies) before being viewed using a Zeiss LSM 880 system. Images were captured using tile-scanning at 25x magnification or no tile-scanning at 40x magnification.

### Laser capture microdissection (LCM)

LCM was performed using a Zeiss PALM MicroBeam system. Diseased transgenic brains were processed as for pathology and then mounted on special MembraneSlide (Zeiss) slides at a thickness of 7 μm. Before LCM capturing, the slides were de-paraffinised using HistoClear II solution (VWR) twice for 5 minutes each. The slides were allowed to dry completely after dehydration in 100% ethanol twice for 10 minutes each. Different areas of interest were captured in special AdhesiveCap tubes (Zeiss). Extraction of genomic DNA was carried with using a QIAamp DNA FFPE Tissue Kit (Qiagen) according to manufacturer’s instructions. For PCR of the recombined (AG2 band) and non-recombined (GFP band) transgene, a previously described method was used^1^. High fidelity amplified bands (Q5 polymerase, NEB) were cut from an agarose gel and purified using Qiagen MinElute kit and cloned into Zero-Blunt TOPO vector (Life Technologies) before transformation into chemically competent DH5α *E*.coli. Three individual clones were selected from Kanamycin plates for each of the AG2 and GFP bands and sequenced using SP6, M13 or T7 primers in the forward and reverse directions. Relevant sequences are shown in Supplementary Fig. 1. Alignment of the sequenced bands with the reference sequence was done using BLAST at NIH.

## Results

**Figure 1:**
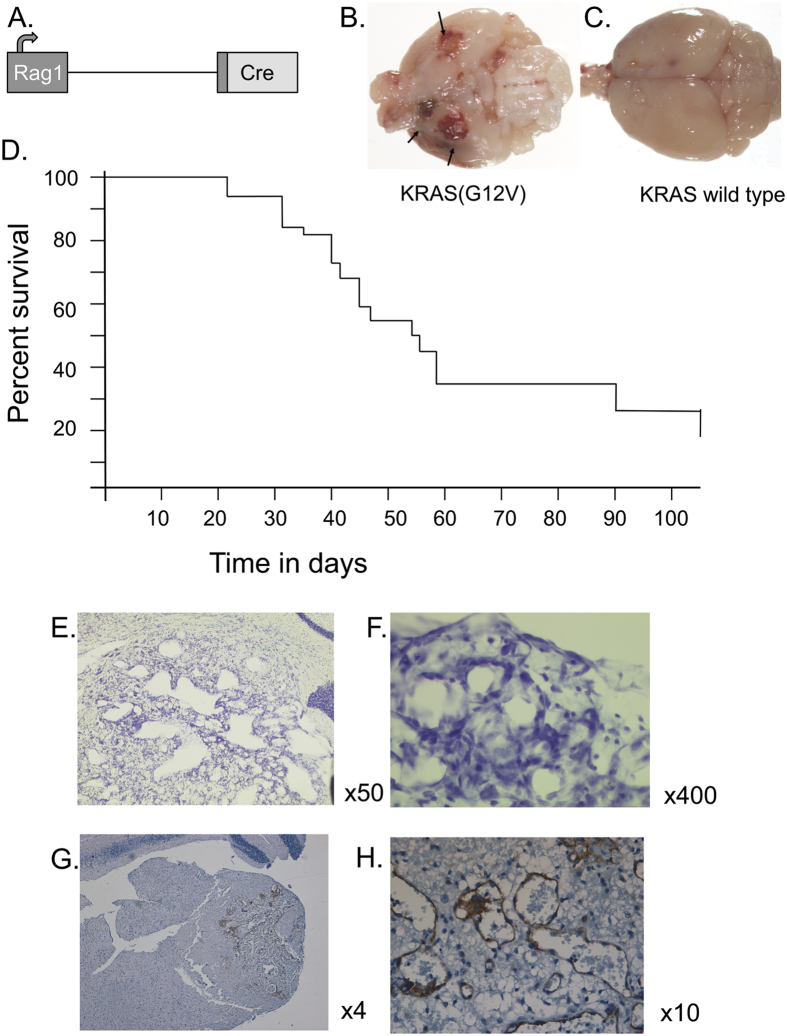
Incidence of haemangioblastoma in mice expressing mutant *Kras.* A cohort of double transgenic *Rag1-Cre*; *KRasG12V* floxed mice (n = 16) were monitored over a period of 120 days (Fig. 1A shows a diagram of *Rag1-Cre* allele^4^ and the flox-STOP *KrasG12V* allele^3^ is illustrated in Fig. 2C). Post-mortem was carried out on individual mice when signs of ill health were found. After sacrifice with fixation perfusion, whole mount brains are shown from 30 day *Rag1-Cre*; *KRasG12V* floxed mice (**B**) and wild type mice (**C**). A Kaplan-Meyer survival curve (**D**) reflects the incidence of disease. No abnormal phenotype was observed in *Rag1-Cre* mice or in *KRasG12V* floxed mice (n = 27) during the experimental period. Brains from affected mice were sectioned and stained with cresyl violet (**E**, x50; **F**, x400) or with haematoxylin (**G**, 4x; **H**, 10x). Sections were immunostained with rabbit anti-mouse CD31 antibody to detect endothelial cells forming irregular vascular channels and pseudocystic spaces, with some interstitial stromal cells, characteristic of haemangioblastomas (**G**, **H**).

We discovered a mouse model of haemangioblastoma caused by activation of a conditional form of floxed mutant Kirsten *Ras (KRas*) with glycine 12 mutation to valine (KRasG12V)^3^ using the VDJ recombinase activating gene 1 (*Rag1*) to express Cre recombinase^4^. Unexpectedly, we observed that the majority of the mice carrying both the floxed *KRasG12V* gene and the *Rag1-Cre* (Fig. 1A) developed an abnormal gait and light sensitivity within two months and post-mortem examination revealed hydrocephalus and CNS vascular proliferations with features of haemangioblastoma. The typical phenotypic features were a tendency to be off balance, moving in circles, unstable gate and sensitivity to light. The brains of these animals showed signs of widespread haemorrhage, predominantly in the cerebrum (Fig. 1B,C).

Interbreeding a mouse strain carrying a conditional allele of *KRASG12V* with a *Rag1-Cre* strain gave rise to double transgenic mice that developed an abnormal gait in ~80% of individuals with a mean onset at 40 days (Fig. 1D). Histologically the brains show vascular tumours (Fig. 1E,F) that have histopathological features typical of human CNS haemangioblastoma., including variable and irregularly sized blood vessel channels and pseudocystic spaces, lined by endothelial cells with pleomorphic nuclei, with accompanying stromal cells. The mouse model CNS tumour lesions showed a close juxtaposition of endothelial lined capillaries type vessels and stromal cells. The stromal cells often had a pink vacuolated appearance indicative of lipid accumulation, but no stromal cell mitotic figures were seen. The vascular channel lining cells were immunostained positively with anti-mouse CD31 antibody (Fig. 1G,H) confirming detection of endothelial cells forming irregular vascular channels and pseudocystic spaces, (with negatively staining interstitial vacuolated stromal cells), characteristic of haemangioblastomas. The tumours expand and infiltrate into the surrounding brain tissue with destructive effects, resulting in adverse brain functions.

**Figure 2:**
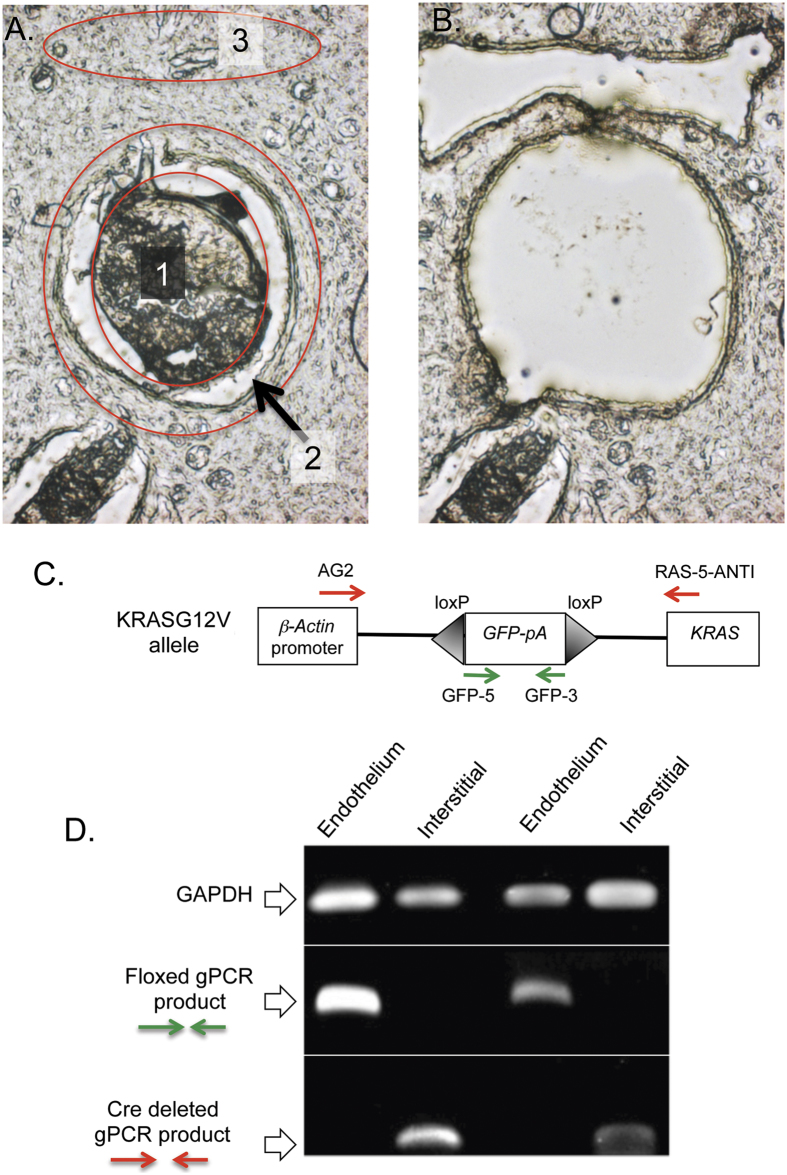
Laser capture microscopy and genomic PCR from brain endothelial cells. The brain from a 30 day old mouse with haemangioblastoma was fixed and sectioned for laser capture microscope recovery of endothelial lesion cells (2) and interstitial cells (3). Panel A and panel B show sections before and after LCM, respectively (1, intra-vascular cells). Genomic DNA was isolated from the cells and subjected to PCR amplification using the published PCR primers^3^ indicated in panel C. PCR products were separated on 1% agarose gels (panel D) using the PCR product of GAPDH as the control for captured DNA. Two independent analyses gave similar results. PCR products were cloned and sequenced; relevant sequences are shown in Supplementary Information Fig. 1. NB: the gels shown in panel D are cropped for illustration purposes and the originals are shown in Supplementary Information Fig. 2.

As Rag1 expression is not associated with blood vessel endothelium, we sought evidence of the Cre-activated *KrasG12V* in isolated endothelial cells using laser capture microscopy (LCM). Endothelial layers from the haemangioblastomas were marked in fixed sections (Fig. 2A) and dissected together with interstitial tissue (Fig. 2A,B shows before & after LCM). Genomic DNA was extracted and PCR carried out with primers that detect the floxed *GFP* gene and the deleted allele (i.e. activated *Kras*) following Cre-mediated deletion (and *Gapdh* as a control) (shown in Fig. 2C). Two separate gDNA LCM isolations were analysed (Fig. 2D), and in both we detected the activated *Kras* allele in the interstitial cells, but not the floxed allele, whereas the floxed allele was still present in the endothelial cells but not the activated *Kras* allele. This suggests that the endothelial proliferation is not cell-intrinsic but rather induced by cells in the surrounding tissue that have acquired activated Kras and presumably secreting excitatory molecules.

**Figure 3:**
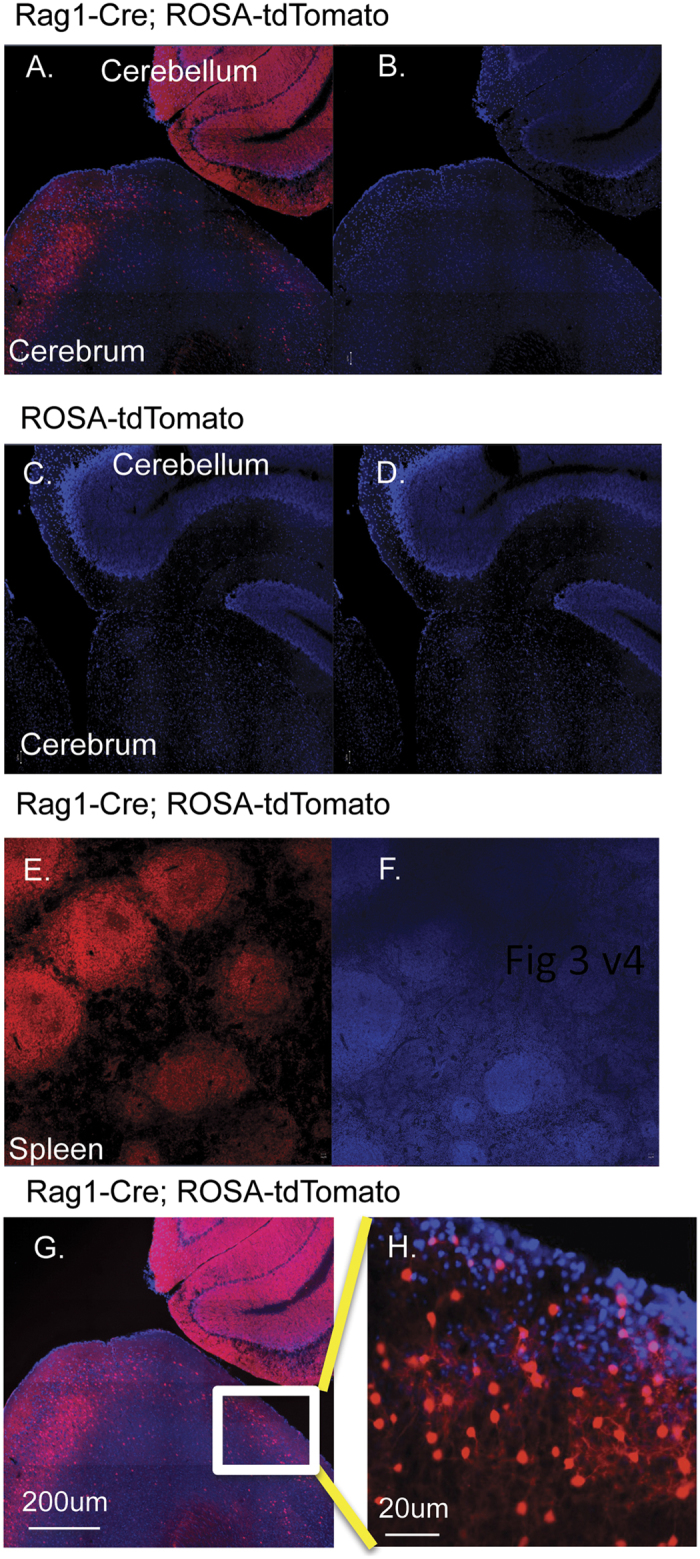
Heterogeneity of *tdTomato* reporter gene expression in *Rag1-Cre* mouse brains. *Rag1-Cre* mice were crossed with *ROSA-tdTomato*^5^ reporter mice and perfusion fixed when 30 days old. Brains were dissected from *Rag1-Cre; ROSA-tdTomato* double positive and from *ROSA-tdTomato-*only mice. Sections were prepared and stained as described in the online methods. Confocal microscope tiled images showing red fluorescence or DAPI stain. Panels A, B; a *Rag1-Cre; ROSA-tdTomato* brain section. Panels C, D; a *ROSA-tdTomato*-only brain section. Panels E, F; *Rag1-Cre; ROSA-tdTomato* spleen section; Panels A, C and E are red fluorescent images overlaid on DAPI stain; panels B, D, F are DAPI-only images. Panels G, H; show the *Rag1-Cre; ROSA-tdTomato* brain section from panel A at higher fluorescence intensity and the boxed region is shown at higher magnification (cerebrum) in panel H. Identical data were obtained from several technical and biological repeats from independent transgenic mating experiments. The size bars are 200 um (panels A–G) and 20 uM in panel H.

Since the endothelial expansion is non-cell autonomous, other cells within the brain must be expressing the *Rag1-Cre* targeted allele. This was examined by employing a reporter mouse strain called *ROSA-tdTomato* that conditionally expresses the red fluorescent protein Tomato^5^. *Rag1-Cre* mice were crossed with *ROSA-tdTomato* mice, and post-mortem heterogeneous cell staining was found in sections of cerebellum and cerebrum of *Rag1-Cre*; *ROSA-tdTomato* double transgenic mice (Fig. 3A,B) but not *ROSA-tdTomato*-only mice (Fig. 3C,D). Spleens of *Rag1-Cre*; *ROSA-tdTomato* double transgenic mice as expected had abundant fluorescent cells (Fig. 3E,F). High power analysis of the cerebrum from the *Rag1-Cre*; *ROSA-tdTomato* double transgenic mouse indicated multiple cell types are present in the brain that express the Tomato reporter (Fig. 3G,H).

## Discussion

The tumours observed in this mouse model represent the comparative pathological equivalent of human CNS haemangioblastomas with marked histological similarity displaying as highly vascular tumours with stromal cells similar to haemangioblastomas in humans. In a genome wide study of human angiosarcoma, RAS mutations (K, H or NRAS) were found in 5 out of 39 cases but other genes were considered to be aetiological^6^. In our model, we show using LCM that the Cre-dependent *KrasG12V* activation does not occur directly in endothelial cells but rather in adjacent cells that express growth factors to drive vascular endothelial proliferation by a paracrine mechanism.

Although the exact identity of these cells is unknown, candidates are CNS cells that have Rag1 expression during development^7^. Cells in the brain that express the *Rag*-*1* gene cause activation of the mutant *Kras* in this mouse model and that are responsible for the angiogenic effects we observe giving stimulus to the endothelial lesions. Astrocytes have been shown to secrete vascular endothelial growth factor (VEGF) in post-natal brains^8^,^9^ and VEGF secretion can be stimulated in cells by activation of mutant *KRAS*^10^. Non-haematopoietic expression of *Rag1* in brain cells remains controversial but our results support the hypothesis that Rag1 is important in brain function. Our work suggests a further study should be undertaken to establish the roles of Rag1 in brain biology and of VEGF-VEGFR interaction in CNS haemangioblastoma. The observation that mutant RAS expressing cells can induce endothelial cell proliferation also has important potential implications for angiogenesis in solid tumour growth where neo-vascularisation enhances tumour growth and supports dissemination of disease to create metastasis. In turn, the metastatic deposits, themselves carrying mutant RAS, could engender neo-vascularisation to sustain their own expansion.

## Additional Information

**How to cite this article:** Bao, L. *et al*. A non-cell autonomous mouse model of CNS haemangioblastoma mediated by mutant KRAS. *Sci. Rep.* **7**, 44899; doi: 10.1038/srep44899 (2017).

**Publisher's note:** Springer Nature remains neutral with regard to jurisdictional claims in published maps and institutional affiliations.

## Supplementary Material

Supplementary Information

## References

[b1] Cushing H. & Bailey P. Hemangiomas of Cerebellum and Retina (Lindau’s Disease): With the Report of a Case. Trans Am Ophthalmol Soc 26, 182–202 (1928). 16692792 PMC1316688

[b2] Neumann H. P. . Central nervous system lesions in von Hippel-Lindau syndrome. J Neurol Neurosurg Psychiatry 55, 898–901 (1992). 1431953 10.1136/jnnp.55.10.898 PMC1015185

[b3] Meuwissen R., Linn S. C., van der Valk M., Mooi W. J. & Berns A. Mouse model for lung tumorigenesis through Cre/lox controlled sporadic activation of the K-Ras oncogene. Oncogene 20, 6551–6558, doi: 10.1038/sj.onc.1204837 (2001). 11641780

[b4] McCormack M. P., Forster A., Drynan L., Pannell R. & Rabbitts T. H. The LMO2 T-cell oncogene is activated via chromosomal translocations or retroviral insertion during gene therapy but has no mandatory role in normal T-cell development. Mol Cell Biol 23, 9003–9013 (2003). 14645513 10.1128/MCB.23.24.9003-9013.2003 PMC309712

[b5] Madisen L. . A robust and high-throughput Cre reporting and characterization system for the whole mouse brain. Nat Neurosci 13, 133–140, doi: 10.1038/nn.2467 (2010). 20023653 PMC2840225

[b6] Behjati S. . Recurrent PTPRB and PLCG1 mutations in angiosarcoma. Nat Genet 46, 376–379, doi: 10.1038/ng.2921 (2014). 24633157 PMC4032873

[b7] Chun J. J., Schatz D. G., Oettinger M. A., Jaenisch R. & Baltimore D. The recombination activating gene-1 (RAG-1) transcript is present in the murine central nervous system. Cell 64, 189–200 (1991). 1986864 10.1016/0092-8674(91)90220-s

[b8] Argaw A. T. . Astrocyte-derived VEGF-A drives blood-brain barrier disruption in CNS inflammatory disease. J Clin Invest 122, 2454–2468, doi: 10.1172/JCI60842 (2012). 22653056 PMC3386814

[b9] Verkhratsky A., Matteoli M., Parpura V., Mothet J. P. & Zorec R. Astrocytes as secretory cells of the central nervous system: idiosyncrasies of vesicular secretion. EMBO J 35, 239–257, doi: 10.15252/embj.201592705 (2016). 26758544 PMC4741299

[b10] Matsuo Y. . K-Ras promotes angiogenesis mediated by immortalized human pancreatic epithelial cells through mitogen-activated protein kinase signaling pathways. Mol Cancer Res 7, 799–808, doi: 10.1158/1541-7786.MCR-08-0577 (2009). 19509115 PMC4267726

