# Supplementary material for: A non-cell autonomous mouse model of CNS haemangioblastoma mediated by mutant KRAS
Source: Sci Rep. 2017 Mar 21;7:44899. doi: 10.1038/srep44899 (PMC5359595; doi:10.1038/srep44899)
Supplement: Supplementary Information [file srep44899-s1.pdf]

A non-cell autonomous mouse model of CNS haemangioblastoma mediated by mutant KRAS

Leyuan Bao, Osama Al-Assar, Lesley F. Drynan, Mark J. Arends, Pam Tyers, Roger A. Barker, Terence H. Rabbitts

Supplementary information files

- Supplementary Figure 1
- Supplementary Figure 2

Supplementary Figure 1

A.

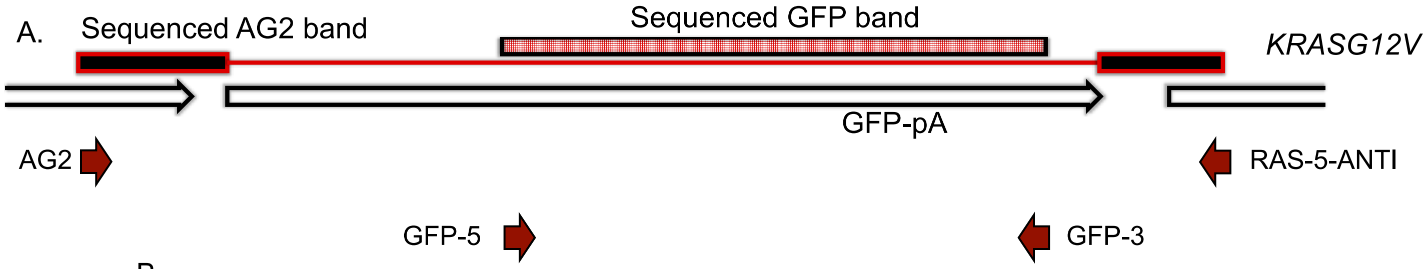

B.

GFP 5’ primer:
5'GACCACATGAAGCAGCACGAC

GFP PCR band sequence:
GACCACATGAAGCAGCACGACTTCTTCAAGTCCGCCATGCCCGAAGGCTACGTCCAG
GAGCGCACCATCTTCTTCAAGGACGACGGCAACTACAAGACCCGCGCCGAGGTGAAG
TTCGAGGGCGACACCCTGGTGAACCGCATCGAGCTGAAGGGCATCGACTTCAAGGAG
GACGGCAACATCCTGGGGCACAAGCTGGAGTACAACTACAACAGCCACAACGTCTATA
TCATGGCCGACAAGCAGAAGAACGGCATCAAGGTGAACTTCAAGATCCGCCACAACAT
CGAGGACGGCAGCGTGCAGCTCGCCGACCACTACCAGCAGAACACCCCCATCGGCG
ACGGCCCCGTGCTGCTGCCCGACAACCACTACCTGAGCACCCAGTCCGCCCTGAGCA
AAGACCCCAACGAGAAGCGCGATCACATGGTCCTGCTGGAGTTCG

C.

AG2 primer:
5'CTGCTAACCATGTTCATGCC

AG2 PCR band sequence:
TCTGCTAACCATGTTCATGCCTTCTTCTTTTTCCTACAGCTCCTGGGCAACGTGCTGGT
TGTTGTGCTGTCTCATCATTTTGGCAAAGATCTTTAATTCAAGCTGGGCGGTCGAGGGA
CCTAGTGAGCAAGGGCGAGGAGCTGTTCACCGGGGTGGTGCCCATCCTGGTCGAGC
TGGACGGCGACGTAAACGGCCACAAGTTCAGCGTGTCCGGCGAGGGCGAGGGCGAT
GCCACCTACGGCAAGCTGACCCTGAAGTTCATCTGCACCACCGGCAAGCTGCCCGTG
CCCTGGCCCACCCTCGTGACCACCCTGACCTACGGCGTGCAGTGCTTCAGCCGCTAC
CCCTGACCGCCGCCGGGATCACTCTCGGCATGGACGAGCTGTACAAGATAACTTCGTA
TAGCATACATTATACGAAGTTATGAATTCGATATCAAGCTTATCGGATGACTGAATATAAAC
TTGTGGTAGTTGGAGCTGTTGGCGTAGG

Figure S1: Sequencing confirmation of LCM PCR products
The organisation of the analysed region of the conditional *KRAS* allele allele is shown in panel A, including the location of the PCR primers (1).
The relevant PCR products shown in text Figure 2 were excised from an agarose gel and cloned into the Topo-zero plasmid for sequence determination.
The sequence shown panel B is from amplification use of primers GFP-5 + GFP-3 and panel C from use of primers AG2 + RAS-5-ANTI

Reference

1. Meuwissen, R., Linn, S.C., van der Valk, M., Mooi, W.J. & Berns, A.
Oncogene **20**, 6551-6558 (2001).

Supplementary Figure 2

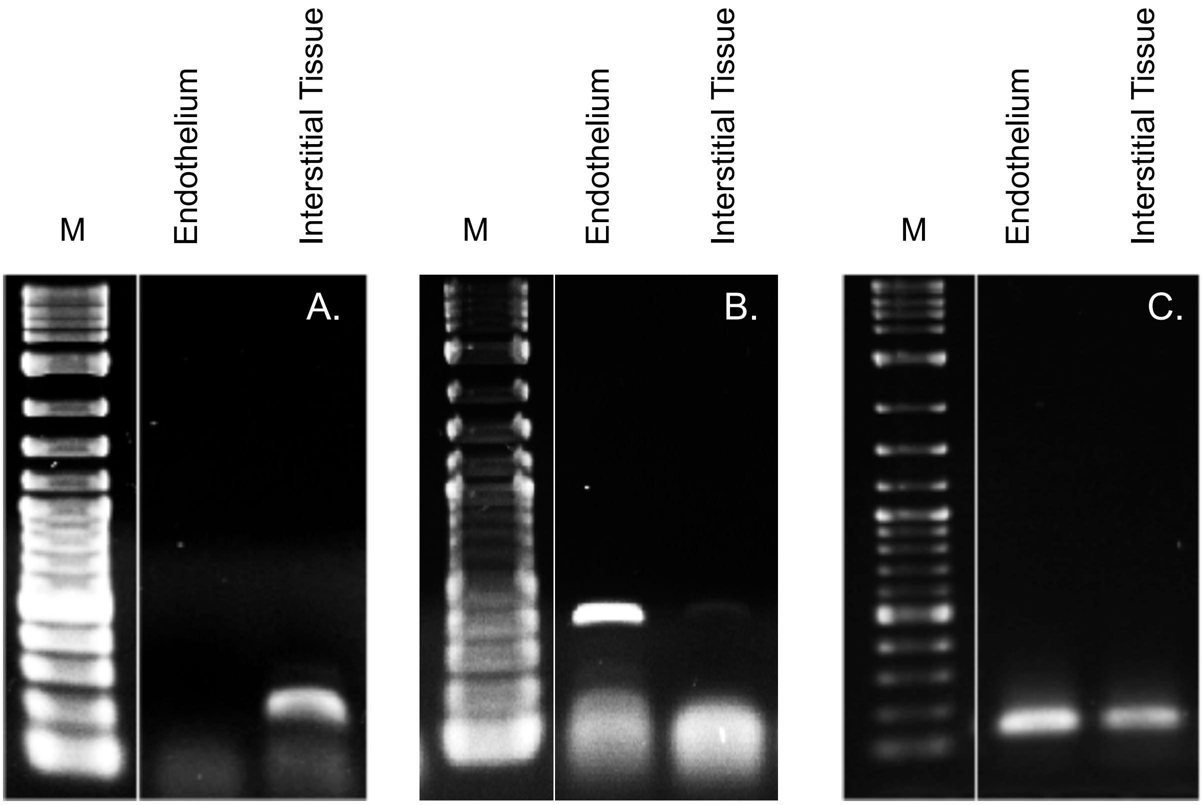

Figure S2: Un-cropped original images of agarose gels of LCM PCR products

Panel A: PCR products using *AG2* + *RAS-5-ANTI* primers
Panel B: PCR products using *GFP5* + *GFP3* primers
Panel C: PCR products using *Gapdh* primers
M = Size marker (2-log ladder New England Biolabs. 0.1 to 10Kb)
